# Multi-cohort analysis of host immune response identifies conserved protective and detrimental modules associated with severity across viruses

**DOI:** 10.1016/j.immuni.2021.03.002

**Published:** 2021-04-13

**Authors:** Hong Zheng, Aditya M. Rao, Denis Dermadi, Jiaying Toh, Lara Murphy Jones, Michele Donato, Yiran Liu, Yapeng Su, Cheng L. Dai, Sergey A. Kornilov, Minas Karagiannis, Theodoros Marantos, Yehudit Hasin-Brumshtein, Yudong D. He, Evangelos J. Giamarellos-Bourboulis, James R. Heath, Purvesh Khatri

**Affiliations:** 1 Institute for Immunity, Transplantation and Infection, School of Medicine, Stanford University, CA 94305, USA; 2 Center for Biomedical Informatics Research, Department of Medicine, School of Medicine, Stanford University, CA 94305, USA; 3 Immunology program, Stanford University, CA 94305, USA; 4 Division of Critical Care Medicine, Department of Pediatrics, School of Medicine, Stanford University, CA 94305, USA; 5 Cancer Biology program, Stanford University, CA 94305, USA; 6 Institute for Systems Biology, Seattle, WA, USA; 7 4^th^ Department of Internal Medicine, National and Kapodistrian University of Athens, Medical School, 124 62 Athens, Greece; 8 Inflammatix, Inc. Burlingame, CA, USA; 9 Department of Bioengineering, University of Washington, Seattle, WA 98195

**Keywords:** pan-virus analysis, conserved host response to viruses, systems immunology, multi-cohort analysis, covid-19

## Abstract

Viral infections induce a conserved host response distinct from bacterial infections. We hypothesized that the conserved response is associated with disease severity and is distinct between patients with different outcomes. To test this, we integrated 4,780 blood transcriptome profiles from patients aged 0 to 90 years infected with one of 16 viruses, including SARS-CoV-2, Ebola, chikungunya, and influenza, across 34 cohorts from 18 countries, and single-cell RNA sequencing profiles of 702,970 immune cells from 289 samples across three cohorts. Severe viral infection was associated with increased hematopoiesis, myelopoiesis, and myeloid-derived suppressor cells. We identified protective and detrimental gene modules that defined distinct trajectories associated with mild versus severe outcomes. The interferon response was decoupled from the protective host response in patients with severe outcomes. These findings were consistent, irrespective of age and virus, and provide insights to accelerate the development of diagnostics and host-directed therapies to improve global pandemic preparedness.

## Highlights

• Conserved host response distinguishes non-severe and severe viral infections
• Increased hematopoiesis, myelopoiesis, and myeloid-derived suppression with severity
• Protective and detrimental modules define trajectories for mild and severe outcomes
• Interferon response is decoupled from the protective response in severe outcomes

## Graphical abstract

**Figure:**
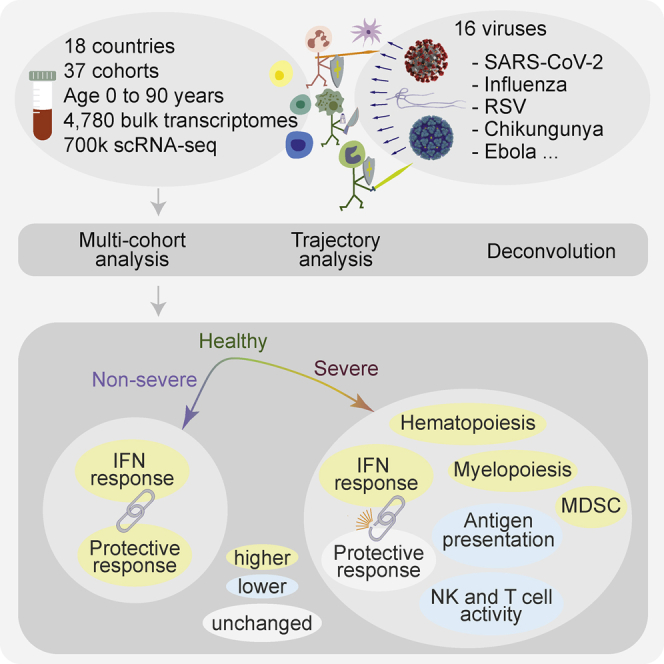

## Teaser

Viral infections induce a conserved host response distinct from bacterial infections, but whether this conserved response distinguishes severity is unclear. Zheng et al. analyzed >5000 bulk and single-cell transcriptome profiles from patients infected with one of 16 viruses, including SARS-CoV-2, Ebola, and chikungunya. They identified protective and detrimental host response modules that distinguish patients with mild or severe outcomes.

## Introduction

Outbreaks of infectious diseases globally have been increasing steadily over the last 40 years (Christiansen, 2018). The first two decades of the 21^st^ century have been marked by seven outbreaks of novel viruses, including severe acute respiratory syndrome coronavirus (SARS-CoV-1), H1N1 influenza, Middle East Respiratory Syndrome Coronavirus (MERS-CoV), chikungunya, Ebola, Zika, and severe acute respiratory syndrome coronavirus 2 (SARS-CoV-2). Four of these outbreaks resulted in pandemics in the last decade (Morens and Fauci, 2020). With each outbreak, a typical approach has been to pursue a pathogen-specific strategy. With novel viruses, when our biological understanding of the causative agent is poor, the acquisition of sufficient knowledge to manage the disease is time-consuming and expensive. Adopting a pathogen-agnostic strategy, such as through the identification of an underlying conserved host response across patient populations, could greatly accelerate the development of diagnostics and therapies to manage future emerging outbreaks. For instance, most approved antiviral drugs are effective against a small number of viruses, and highly susceptible to resistance (Bekerman and Einav, 2015). In contrast, identifying conserved host biology, such as host proteins required by multiple viruses, could be used to develop broad-spectrum antivirals. Similarly, a conserved host response to viral infections could be used to develop diagnostics and prognostics. Several studies have repeatedly demonstrated the utility of the host immune response to pathogens to accurately diagnose the presence and type of infections (Andres-Terre et al., 2015; Sweeney et al., 2015; 2016b; Mayhew et al., 2020). We have previously identified a conserved host response to distinguish bacterial and viral infections (Andres-Terre et al., 2015; Sweeney et al., 2015; 2016b). We have also demonstrated that the conserved host immune response to infection is detected earlier than symptom onset (Andres-Terre et al., 2015; Sweeney et al., 2016a; Warsinske et al., 2018; Gupta et al., 2020; Turner et al., 2020).

Here, we hypothesized that our previously described conserved host response signature to respiratory viral infections, called the Meta-Virus Signature (MVS) (Andres-Terre et al., 2015), is also conserved in viral infections that cause severe disease, including Ebola, SARS-CoV-2, and others, and it could be used to identify common genes associated with detrimental and protective host immune responses, irrespective of the virus. We tested these hypotheses by integrating 34 independent cohorts comprising 4,780 blood transcriptome profiles and single-cell RNA-seq profiles of 702,970 immune cells from 289 samples from healthy controls (HCs) and patients with acute viral infection. We found that the MVS is (1) present in SARS-CoV-2, Ebola, chikungunya, influenza, and other viruses, (2) correlated with severity, and (3) predominantly expressed in myeloid cells. Using a patient trajectory differentiation method, we found that patients with mild or severe viral infection follow different trajectories comprised of four gene modules corresponding to protective and detrimental host immune responses. We defined the severe-or-mild (SoM) score that accurately distinguished patients with non-severe and severe outcomes. By leveraging the biological, clinical, and technical heterogeneity across data, we provide strong evidence of a conserved host immune response to acute viral infection, irrespective of the virus. Further analysis of these conserved host response modules could lead to the development of diagnostics, prognostics, and host-directed therapies for a broad spectrum of viruses that could facilitate risk stratification and targeted treatment of patients during the current pandemic and in novel outbreaks that will inevitably arise in the future.

## Results

### Data collection, curation, and preprocessing

**Figure 1:**
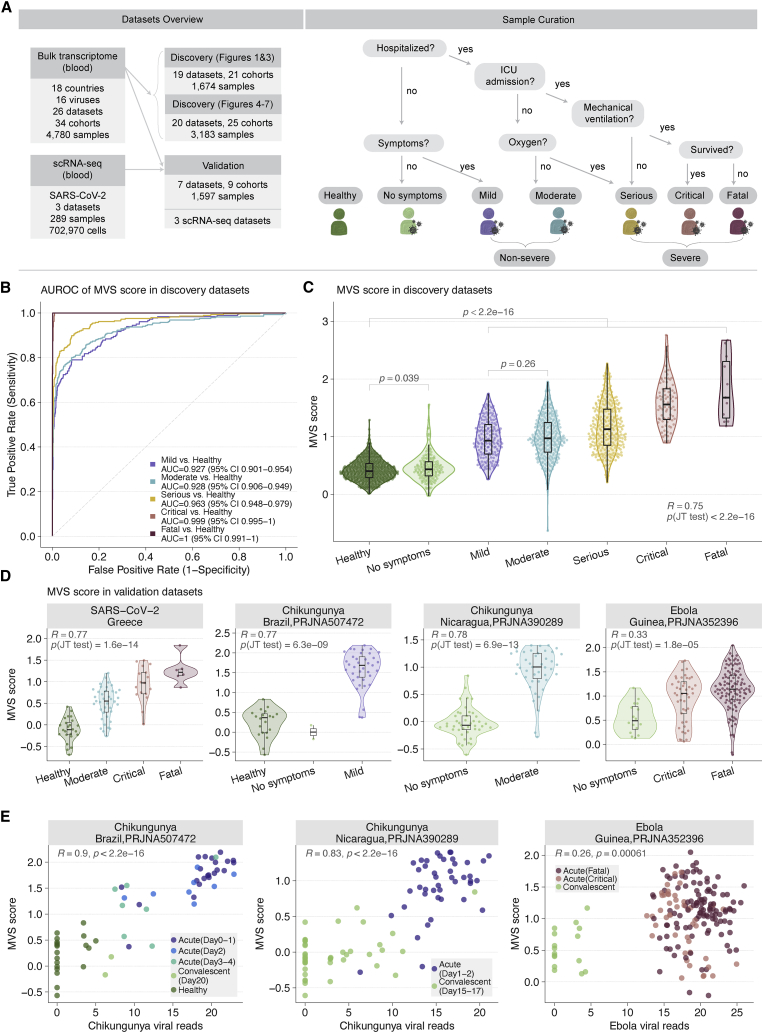
Conserved host response to viral infection, represented by the MVS, is associated with severity (A) Datasets used for analysis divided into discovery and validation (left) and criteria for assigning viral infection severity categories to samples (right). “No symptoms” includes individuals with asymptomatic viral infection or convalescents. (B) ROC curves for distinguishing patients with viral infection of varying severity from HCs using the MVS score (1,674 samples in 19 datasets). (C) Distribution of the MVS scores across the severity of viral infection (1,674 samples in 19 datasets). Each point represents a blood sample. Jonckheere-Terpstra (JT) trend test was used to assess the significance of the trend of the MVS score over severity. p values using Mann–Whitney U test for the comparison of MVS scores in two groups. (D) Validation of correlation between the MVS score severity of viral infection in 4 independent RNA-seq datasets from patients with SARS-CoV-2, chikungunya, or Ebola infection. (E) Positive correlation between the MVS score and the number of viral reads detected in blood samples RNA-seq datasets. Each point represents a sample. The x axis represents the number of viral reads; the y axis represents the MVS score for each sample. p values were computed using Pearson correlation test. See also Figure S1.

We searched the public repositories for blood transcriptome profiles from patients with viral infection (STAR Methods). After excluding datasets used to discover the MVS previously, we identified 26 datasets composed of 4,780 samples from patients across 18 countries infected with at least one of 16 viruses (Figure 1A, Table S1, and Data S1). Overall, these datasets included a broad spectrum of biological, clinical, and technical heterogeneity represented by blood samples profiled from children and adults infected with a virus using either microarray or RNA sequencing. We assigned a standardized severity category to each of the 4,780 samples (Figure 1A and STAR Methods). Briefly, we divided non-hospitalized samples into “no symptoms” or “mild,” and hospitalized patients into “moderate,” “serious,” “critical,” and “fatal” categories based on the level of care required and outcomes as described in the original publications (Figure 1A and STAR Methods). We also defined two broader categories: “non-severe,” encompassing patients with mild and moderate viral infection, and “severe,” encompassing patients with serious, critical, and fatal viral infection (Figure 1A). For cohorts that lacked sample-level severity data, we assigned the same severity category to each sample based on the cohort description.

### MVS represents a conserved host response to viral infection and is associated with severity

To test our hypothesis that a conserved host response to viral infection is associated with severity, we co-normalized 1,674 blood transcriptomes (663 HCs, 167 asymptomatic/convalescent, 181 mild, 286 moderate, 286 serious, 80 critical, and 11 fatal) from 19 independent datasets, the majority of which were infected with adenovirus, influenza, human rhinovirus (HRV), or respiratory syncytial virus (RSV), using COCONUT (Figure 1A, Table S1; STAR Methods) (Sweeney et al., 2016b). The MVS score accurately distinguished patients with viral infection from HCs across all datasets (Figure 1B, Figure S1A) and correlated with severity (r = 0.75, p < 2.2e-16; Figure 1C). The MVS score was higher in all infected patients compared to HCs (p < 2.2e-16), regardless of symptoms, severity, and virus (Figure 1C). In asymptomatic or convalescent patients, the MVS score was marginally higher than in HCs (p = 0.039), but not different between patients with mild versus moderate severity (p = 0.26). Across all datasets, the MVS score was correlated with viral infection severity (0.43 ≤ R ≤ 0.93), regardless of virus, geography, or age (Figure S1B). In 405 samples from patients infected with SARS-CoV-2, Ebola, or chikungunya across 4 datasets, the MVS score was correlated with severity (0.33 ≤ R ≤ 0.78; p ≤ 1.8e-05; Figure 1D) and distinguished patients with viral infection from HCs (Figure S1C). In three independent datasets of blood samples from patients with either chikungunya or Ebola infection, profiled using RNA-seq, we detected sequencing reads from the corresponding viral RNA (STAR Methods). In each of these three datasets, the MVS score significantly correlated with the number of viral reads detected in blood (p ≤ 6.1e-4; Figure 1E). Further, in each dataset, both the number of viral reads in blood and the MVS score decreased as patients progressed from acute infection to convalescence.

Collectively, our results show that a conserved host response to viral infection, represented by the MVS score, is correlated with the severity and the number of viral reads detected in blood samples, irrespective of biological, clinical, or technical heterogeneity or the infecting virus.

### Myeloid cells are the primary source of the MVS

Next, we investigated whether the MVS score is associated with specific immune cell types. We integrated three single-cell RNA-seq (scRNA-seq) datasets consisting of 702,970 immune cells from 289 PBMC samples (258 SARS-CoV-2, 27 healthy, 2 influenza, 2 RSV) from 170 individuals across three independent cohorts (Seattle, Atlanta, Stanford) (Table S2) (Arunachalam et al., 2020; Su et al., 2020; Wilk et al., 2020). The Seattle Cohort profiled 557,240 immune cells from 258 PBMC samples of HCs and patients with SARS-CoV-2 infection (16 healthy, 94 asymptomatic, 8 mild, 37 moderate, 78 serious, 21 critical, 3 fatal) using CITE-seq. The patients with SARS-CoV-2 infection in the Seattle Cohort were profiled at two time points: (1) near the time of a positive clinical diagnosis and (2) a few days later. The Atlanta Cohort profiled 76,929 immune cells from 18 PBMC samples of HCs and patients infected with one of 3 viruses (5 healthy, 1 moderate influenza, 1 serious influenza, 2 serious RSV, 2 convalescent SARS-CoV-2, 3 moderate SARS-CoV-2, 3 serious SARS-CoV-2, 1 fatal SARS-CoV-2) using CITE-seq. Finally, the Stanford Cohort profiled 68,801 immune cells from 13 PBMC samples of HCs and patients with SARS-CoV-2 infection (6 healthy, 1 moderate, 3 serious, 2 critical, 1 fatal) using Seq-Well. Collectively, these three cohorts included clinical, biological, and technical heterogeneity at a single-cell level.

**Figure 2:**
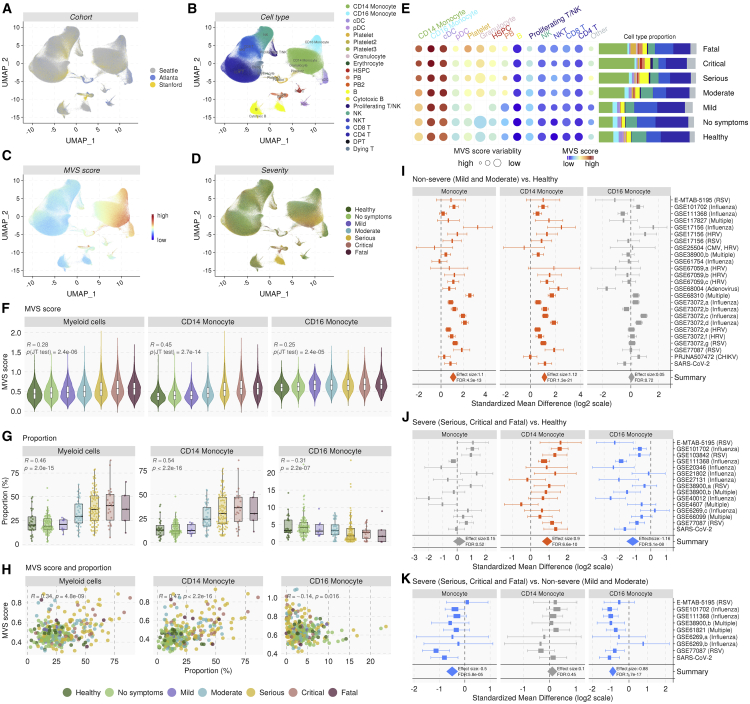
Single-cell RNA-seq identifies monocytes as the primary source of the MVS (A–D) UMAP visualization of 702,970 immune cells from 289 PBMC samples in three independent scRNA-seq datasets colored by (A) cohort, (B) cell type, (C) MVS score, and (D) severity of viral infection. (E) Circle map depicting the average MVS score in each cell type in each severity category, where color represents the average MVS score in each cell type, and size is proportional to the variability of MVS score in the cell type with larger size representing lower variability. The barplot shows the mean proportion of each cell type in each severity category. (F) MVS score in in myeloid cells, CD14+, and CD16+ monocytes. p values were computed using JT trend test. (G) Proportion of myeloid cells, CD14+, and CD16+ monocytes in each sample. p values were computed using JT trend test. (H) Correlation between the MVS score and proportion of myeloid cells, CD14+ and CD16+ monocytes. p values were computed using Pearson correlation test. (I–K) Changes in proportions of total, CD14+, and CD16+ monocytes estimated using *in silico* deconvolution of bulk transcriptome profiles. Forest plots for change in proportions of total, CD14+, and CD16+ monocytes in (I) non-severe patients versus HCs, (J) severe patients versus HCs, and (K) severe versus non-severe patients. The x axes represent standardized mean difference between two groups, computed as Hedges’ *g*, in log2 scale. The size of a rectangle is proportional to the standard error of mean difference in the study. Whiskers represent the 95% confidence interval. The diamonds represent overall, combined mean difference for a given cell type in a given comparison. Width of the diamonds represents the 95% confidence interval of overall mean difference. See also Figure S2.

We integrated the three scRNA-seq cohorts using Seurat (Satija et al., 2015) (Figures 2A–2D, Figures S2A–S2C). Immune cells across the three cohorts clustered into myeloid cells (monocytes, myeloid dendritic cells, granulocytes, etc.), T and NK cells, and B cells (Figures 2A and 2B, Figure S2A). The MVS score was substantially higher in myeloid cells from hospitalized patients with viral infection (Figures 2C–2E, Figures S2B and S2C) and positively correlated with the severity of viral infection in myeloid cells (R = 0.28, p = 2.4e-06), which was driven by CD14+ monocytes (R = 0.45, p = 2.7e-14) compared to CD16+ monocytes (R = 0.25, p = 2.4e-05) (Figure 2F). Further, proportions of myeloid cells increased with severity (R = 0.46, p = 2.0e-15), which was also driven by CD14+ monocytes (R = 0.54, p < 2.2e-16). Proportions of CD16+ monocytes decreased with increasing severity of viral infection (R = −0.31, p = 2.2e-07) (Figure 2G). The MVS score in myeloid cells at the single-cell level and proportions of myeloid cells were positively correlated (R = 0.34, p = 4.8e-09), which was also driven by CD14+ monocytes (R = 0.47, p < 2.2e-16) (Figure 2H). Together, these results showed that in response to a viral infection, proportions of and the conserved host response to viral infection at a single-cell level in CD14+ monocytes increase with severity.

Next, we performed *in silico* cellular deconvolution of blood transcriptome profiles of 4357 patient samples from 32 independent cohorts using immunoStates (Bongen et al., 2018; Chowdhury et al., 2018; Scott et al., 2019; Vallania et al., 2018) (Table S3) to investigate if these changes in CD14+ and CD16+ monocytes were also observed at the bulk transcriptome level. We performed three multi-cohort analyses to compare changes in estimated immune cell proportions in (1) non-severe viral infections compared to HCs, (2) severe viral infections compared to HCs, and (3) severe compared to non-severe viral infections.

Similar to scRNA-seq analysis, proportions of total monocytes were significantly higher in patients with non-severe viral infection compared to HCs (ES = 1.10, FDR = 4.33e-13), but not in those with severe viral infection (Figures 2I–2J, Table S4). The proportion of CD14+ monocytes increased significantly in patients with non-severe (ES = 1.12, FDR = 1.30e-21) and severe (ES = 0.9, FDR = 6.56e-10) viral infection compared to HCs, but were not different between patients with non-severe or severe viral infection (Figures 2I–2K, Table S4). In line with the scRNA-seq data, proportions of CD16+ monocytes were significantly lower in patients with severe viral infection compared to HCs (ES = −1.16, FDR = 5.13e-08) and those with non-severe viral infection (ES = −0.88, FDR = 1.73e-17) (Figures 2J–2K, Table S4), but were unchanged in non-severe patients compared to HCs (Figure 2I, Table S4).

Cellular deconvolution analysis also found that the proportions of neutrophils were significantly higher in patients with severe viral infection compared to HCs (ES = 1.24, FDR = 4.12e-16) and those with non-severe viral infection (ES = 0.99, FDR = 4.33e-07) (Figure S2D**,** Table S4). These cells are likely low-density immature granulocytes typically found in patients with sepsis.

Collectively, our integrated analyses of 4646 samples across 35 independent cohorts using scRNA-seq and *in silico* deconvolution of bulk transcriptome profiles showed that the conserved host response to viral infections is predominantly from myeloid cells, where proportions of CD14+ monocytes increased and CD16+ monocytes decreased with increased severity of viral infection.

### MVS identifies distinct clusters of patients with non-severe and severe viral infection

**Figure 3:**
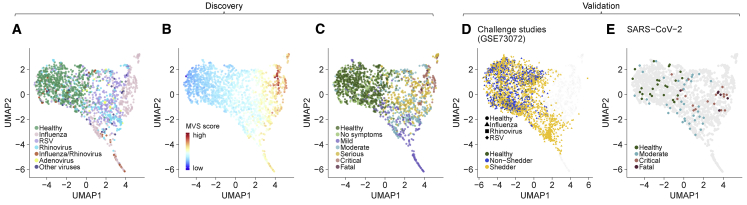
The MVS identifies distinct clusters of patients with non-severe and severe viral infection (A–C) UMAP visualizations of the discovery cohorts (1,674 samples in 19 datasets) colored by (A) virus (B) MVS score, and (C) severity of viral infection. (D and E) Projection of independent cohorts on the UMAP space obtained from the discovery cohorts: (D) seven challenge studies using influenza, RSV, or HRV in GSE73072 (2,518 samples) and (E) the SARS-CoV-2 cohort (86 samples).

As expected, low dimensional visualization of 1674 co-normalized samples using UMAP showed that HCs were distinct from patients with viral infection irrespective of the infecting virus (Figure 3A). The MVS score increased along the first UMAP component (UMAP1; Figure 3B), while patients with mild viral infection were clustered separately from those with severe viral infection along the second UMAP component (UMAP2; Figure 3C). We further validated the robustness of the localization of samples by severity observed in UMAP by mapping 8 independent cohorts consisting of 2,604 samples from patients with one of 4 viral infections to the same low dimensional space (Figures 3D and 3E). All but one of these 8 cohorts were challenge studies, where 129 healthy individuals were inoculated with influenza (1,465 samples from 70 subjects), RSV (419 samples from 20 subjects), or HRV (634 samples from 39 subjects). Each of the infected subjects in these challenge studies had asymptomatic or mild infection. When mapped to the UMAP space created using the 1,674 samples, samples from the challenge studies clustered with mild viral infections (Figure 3D). In contrast, patients with critical and fatal SARS-CoV-2 infection mapped to the region enriched for patients with critical and fatal viral infection, whereas patients with moderate SARS-CoV-2 infection mapped to the region enriched for patients with non-severe viral infection (Figure 3E), again demonstrating that the host response to viral infection is conserved and associated with severity, irrespective of the virus. Together, this observation suggested that a distinct subset of genes in the MVS may be differentially associated with the severity of viral infection.

### Hospitalized patients with viral infection follow a different trajectory from non-hospitalized patients with viral infection

Based on the UMAP of samples, we hypothesized that patients with mild and severe viral infection follow different trajectories. Analogous to cellular differentiation analysis using single-cell profiling data, where each cell represents a snapshot along the differentiation trajectory, each sample in our analysis represents a snapshot of the host response to viral infection that spans from recognizing the presence of a virus to its elimination. To test this hypothesis, we adapted tSpace (Dermadi et al., 2020), a method for identifying cellular differentiation trajectories using single-cell data, to identify disease trajectories using bulk RNA data. We refer to the modified method as “disease space” (dSpace) (STAR Methods).

We co-normalized four of the seven randomly selected challenge studies (1,509 samples across 2 influenza, 1 HRV, and 1 RSV studies) with 1674 samples from 19 datasets using COCONUT to leverage a large number of longitudinal samples that can aid in a more accurate inference of the host response trajectories. Overall, we applied dSpace to 3,183 COCONUT co-normalized samples (1,663 HCs, 343 no symptoms, 514 mild, 286 moderate, 286 serious, 80 critical, 11 fatal) from 25 independent cohorts. We used these left-out challenge studies for validation of the inferred trajectories and to avoid possibility of introducing class imbalance because subjects in the challenge studies only had mild viral infections.

**Figure 4:**
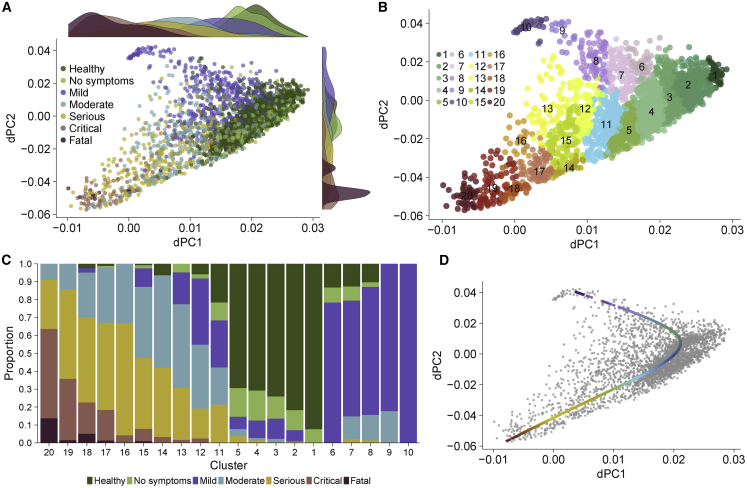
Patients with non-severe and severe viral infection follow divergent disease trajectories (A) Trajectory analysis using dSpace (3,183 samples in 25 cohorts). (B) Clustering of samples using dSpace. (C) Proportion of samples for each severity category in each cluster. (D) A principal line on dSpace coordinates identified by trajectory analysis. The red and purple colors of the line ends indicate the severe and non-severe trajectories, respectively. See also Figure S3.

The first principal component of dSpace (dPC1) correlated with the severity of viral infection, and the second component (dPC2) distinguished hospitalized patients with viral infection from non-hospitalized patients with mild infection (Figure 4A). Importantly, participants from the influenza, RSV, and HRV challenge studies clustered almost exclusively with patients with mild infection (Figure S3A).

Next, we clustered samples using the disease space matrix, and used the resulting clusters to isolate trajectories associated with the severity of viral infection (STAR Methods). Clustering the samples using the dSpace matrix identified 20 clusters such that one category of samples dominated a cluster (Figure 4B): clusters 1–5, in which HCs and asymptomatically infected or convalescent patients accounted for >80% of samples; clusters 6–10, in which patients with mild viral infection accounted for >68% of samples, and clusters 13–20, in which hospitalized patients with moderate, serious, critical, or fatal viral infection accounted for >77% of samples (Figure 4C). Clusters 11 and 12 were heterogeneous as no one group of samples dominated them. Out of 1,509 samples, 1,507 (99.9%) from the influenza, RSV, and HRV challenge studies fell within clusters 1–12 (Figure S3B), demonstrating the robustness of the clusters defined using dSpace. We fit a principal trajectory line to the dSpace matrix, which consisted of healthy patients in the center and two divergent trajectories: one dominated by patients with mild viral infection and the other dominated by hospitalized patients with viral infection (Figure 4D, STAR Methods). Hitherto, we refer to these trajectories as “mild trajectory” and “severe trajectory,” respectively. Together, trajectory analysis using dSpace showed that hospitalized patients with viral infection follow a different trajectory than those with mild infection compared to HCs, irrespective of the infecting virus.

### Proportions of NK cells and the expression of NK cell-specific genes negatively correlate with the severity of viral infection

**Figure 5:**
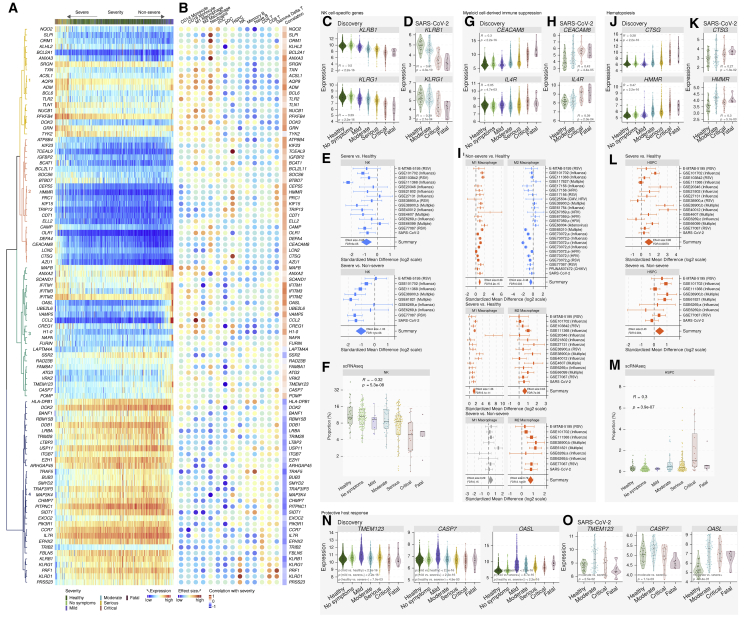
Immune responses from NK cells, myeloid cell-derived suppression, and hematopoiesis are associated with severity of viral infection Violin plots: each dot represents a sample, and the y axis represents expression of the corresponding gene in a sample. p values were computed using Spearman rank correlation test or Mann–Whitney U test. Boxplots: each dot represents a sample, and the y axis represents proportion of the corresponding cell type in a sample. p values were computed using JT trend test. Forest plots: change in proportions between two groups for a given immune cell type, obtained by *in silico* deconvolution, where the x axis represents standardized mean difference (Hedges’ *g*) between two groups in log2 scale. The size of a rectangle is proportional to the standard error of mean difference in the study. Whiskers represent the 95% confidence interval. The diamonds represent summary mean difference for a given cell type in a given comparison. Width of a diamond represents the 95% confidence interval of overall mean difference. (A) Expression heatmap of the 96 trajectory-defining genes. Rows represent genes and columns represent samples, ordered by position along the disease trajectory. Colors of the dendrograms indicate gene modules. (B) For each gene, effect size (Hedge’s *g*) in a given cell type compared to all other cell types and correlation with severity. (C and D) Expression of NK cell-specific genes in (C) the discovery (3,183 samples in 25 cohorts) and (D) a validation cohort (24 HCs, 62 SARS-CoV-2). (E) Change in proportions of NK cells in patients with severe (top panel) and non-severe viral infection (bottom panel) compared to HCs. (F) Proportions of NK cells along the severity of viral infection in three independent scRNA-seq cohorts. (G and H) Expression of *CEACAM8* and *IL4R* in peripheral blood samples from patients with viral infection in (G) the discovery (3,183 samples in 25 cohorts) and (H) a validation cohort (24 HCs, 62 SARS-CoV-2). (I) Change in proportions of pro-inflammatory macrophages (M1) and anti-inflammatory macrophages (M2) in patients with non-severe or severe viral infection compared to HCs. (J and K) Expression of HSPC-specific genes in patients with viral infection in (J) the discovery (3,183 samples in 25 cohorts) and (K) a validation cohort (24 HCs, 62 SARS-CoV-2). (L) Change in proportions of HSPCs patients with severe viral infection compared to HCs (top panel) and non-severe viral infection (bottom panel). (M) Proportions of HSPCs along the severity of viral infection in three independent scRNA-seq cohorts. (N and O) Genes with higher expression in patients with mild or moderate viral infection compared to HCs and those with severe viral infection in (N) the discovery and (O) a validation cohort. See also Figures S4 and S5.

We identified 96 genes within the MVS that were significantly different between the two trajectories (Figure 5A). Using the MetaSignature database (https://metasignature.stanford.edu), we found the majority of the genes negatively correlated with the severity of viral infection are preferentially expressed in lymphocytes (T cells, B cells, and NK cells), whereas the majority of the genes positively correlated with severity are preferentially expressed in myeloid cells (granulocytes, monocytes, mDCs, and macrophages) (Figure 5B) (Haynes et al., 2017; Vallania et al., 2018).

Several NK cell-specific genes from the killer cell lectin-like receptor (KLR) family (*KLRB1, KLRG1, KLRD1*) and phosphoinositide-3-Kinase (PI3K) signaling genes (*PIK3R1*) were negatively correlated with severity (Figure 5C and Figure S4A). These genes were also lower in critical and fatal SARS-CoV-2 infections compared to HCs (Figure 5D, Figure S4B). PI3K signaling in NK cells and mutations in *PIK3R1* have been linked with human immunodeficiency and viral infections (Mace, 2018). Therefore, we hypothesized that NK cell proportions decreased with increased severity of viral infection. Deconvolution of bulk transcriptome showed the proportions of NK cells were significantly lower in patients with severe viral infections compared to HCs (ES = −0.85, FDR = 8.97e-05) and non-severe viral infections (ES = −1.03, FDR = 1.13e-06) (Figure 5E, Table S4). Further, across the three scRNA-seq cohorts, NK cell proportions were inversely correlated with severity (Figure 5F). Together, trajectory analysis using dSpace, deconvolution using immunoStates, and scRNA-seq found that the proportions of NK cells and the expression of NK cell-associated genes reduced with increased severity of viral infection, irrespective of the infecting virus.

### Patients with severe viral infection show reduced antigen presentation

Trajectory analysis identified *HLA-DPB1*, a key HLA class II gene expressed in antigen presenting cells, was negatively correlated with severity, including in patients with SARS-CoV-2 infection (Figure 5A, Figures S5A and S5B). Downregulation of HLA class II in severe COVID-19 patients has been reported previously (Wilk et al., 2020). Expression of *HLA-DPB1* was lower in several antigen presenting cells (CD14 monocytes, conventional dendritic cells (cDCs), and B cells) in patients with moderate and severe viral infection in three scRNA-seq cohorts (Figures S5C and S5D) along with a reduced proportion of cDCs in severe viral infections in both scRNA-seq (Figure S5E) and bulk transcriptome deconvolution (Table S4). Together, our results showed that expression of HLA class II genes is negatively correlated with viral infection.

### Myeloid-derived immune suppression is higher in patients with severe viral infection

Several differentially expressed genes between the two trajectories (Figure 5A) were preferentially expressed by immune cells of the myeloid lineage (Figure 5B). Although a subset of positively correlated genes with viral infection severity (*CAMP*, *BCAT1*, *LCN2*, *TXN*) have known proinflammatory functions in myeloid cells (Bertini et al., 1999; Bruns et al., 2015; Choi and Fujii, 2019; Eriksson et al., 2017; Papathanassiu et al., 2017; Ramos-Martínez et al., 2018) (Figure S4C**)**, we found strong evidence of increased myeloid cell-derived immune suppression in patients with severe viral infection. Markers of polymorphonuclear myeloid-derived suppressor cells (PMN-MDSCs), *CEACAM8* (*CD66B*; Figure 5G) and *OLR1* (*LOX-1;* Figure S4C), and markers of monocytic MDSCs (M-MDSCs), *IL-4R* (Figure 5G), *ITGAM* (*CD11B;* Figure S4D), including a functional marker of MDSCs, *ARG1* (Figure S4D), were positively correlated with the severity of viral infection. *ORM1*, which drives the differentiation of monocytes to anti-inflammatory M2b macrophages (Nakamura et al., 2015), was significantly different between the two trajectories. Genes known to reduce the type I interferon (IFN) response, *GRN* and *BCL6* (Wei et al., 2019; Wu et al., 2016), were positively correlated with severity (Figure S4C). All genes but *GRN* positively correlated with severity in the independent cohort of patients with SARS-CoV-2 infection (Figure 5H, Figure S4D). Notably, *ORM1* expression was lower in mild patients but higher in severe patients compared to HCs, including SARS-CoV-2-infected patients (Figures S4C and S4D).

Therefore, we hypothesized that proportions of pro- and anti-inflammatory macrophages would differ between patients with non-severe versus severe viral infection. Deconvolution analysis showed proportions of pro-inflammatory (M1) macrophages were higher in patients with non-severe (ES = 0.88, FDR = 6.16e-15) and severe (ES = 1.36, FDR = 5.12e-11) viral infection compared to HCs (Table S4), whereas proportions of anti-inflammatory (M2) macrophages were lower in non-severe patients (ES = −0.48, FDR = 3.00e-03), but higher in severe patients compared to HCs (ES = 0.63, FDR = 7.02e-06) and non-severe patients (ES = 0.76, FDR = 3.12e-09). Together, we found strong evidence of increased myeloid-derived immune suppression in patients with severe viral infection.

### Increased hematopoiesis in patients with severe viral infection

Several differentially expressed genes between the two trajectories (*CTSG*, *PRC1*, *DEFA4, KIF15, TCEAL9, HMMR, CEP55,* and *AZU1*) were overexpressed in patients with severe viral infection, but not in those with non-severe viral infection compared to HCs (Figures 5J–5K, Figures S4E and S4F). All but one of these genes (*DEFA4*) have higher expression in circulating HSPCs (Figure 5B). Therefore, we investigated whether HSPCs were higher in patients with severe viral infection, but not in those with non-severe viral infection. Deconvolution analysis found that HSPCs were significantly higher in patients with severe viral infection compared to HCs (ES = 0.85, FDR = 7.33e-04) and compared to patients with non-severe viral infection (ES = 0.43, FDR = 3.38e-02) (Figure 5L, Table S4), but not in those with non-severe viral infection compared to HCs (Table S4). Proportions of HSPCs increased with severity in scRNA-seq across three independent cohorts of SARS-CoV-2-infected patients (Figure 5M).

### Trajectory analysis identifies a protective host response associated with mild viral infections

Finally, dSpace analysis identified several genes (*CCL2*, *OASL, CASP7, TMEM123, MAFB, VRK2, UBE2L6, NAPA*) higher in patients with mild viral infection than those with severe viral infection or HCs (Figures 5N and 5O, Figures S4G and S4H). *CCL2,* a type I IFN receptor-mediated chemoattractant that promotes monocyte migration to the site of infection, and *OASL*, a type I IFN-induced gene, had higher expression in patients with mild viral infection. *CASP7* is cleaved by *CASP3* and *CASP10* and is activated upon cell death stimuli and induces apoptosis. *TMEM123* (*PORIMIN*) is a cell surface receptor that mediates oncosis, a type of cell death distinct from apoptosis characterized by a loss of cell membrane integrity without DNA fragmentation. Together, these results suggest that patients with a coordinated immune response involving monocyte recruitment, IFN response, and higher cell death have a lower risk of severe viral infection.

### Protective and detrimental host response modules are associated with the severity of viral infection

Unsupervised hierarchical clustering grouped the 96 genes into four modules (Figure 5A). Module 1 and 2 were composed of genes preferentially expressed in myeloid and HSPCs and were higher in patients with severe viral infection (Figure 5B). Module 4 was composed of genes preferentially expressed in lymphoid cells (NK, T, and B cells). Genes in module 3 and 4 were higher expressed in patients with mild viral infection compared to those with severe infection (Figure 5B). These four modules broadly divided the host response genes differentially expressed between two trajectories into two categories: a detrimental host response represented by module 1 and 2 (higher in patients with severe viral infection), and a protective host response represented by module 3 and 4 (higher in patients with mild viral infection).

**Figure 6:**
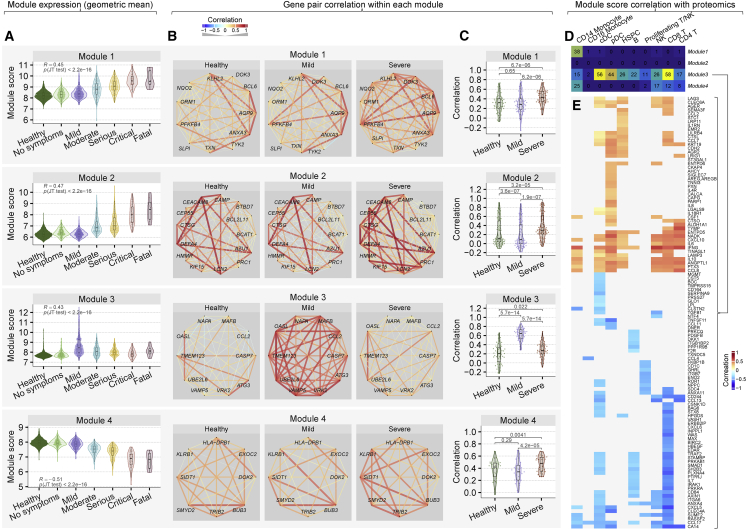
Coordinated protective and deleterious host response modules associated with severity of viral infection (A) Module scores, defined as the geometric mean of expression of genes in each module in the sample, for the discovery cohorts (3,183 samples in 25 cohorts). (B) Pairwise Spearman’s rank correlation coefficient between genes in each module in HCs and patients with mild or severe viral infection. The width of the line indicates strength of correlation; red and blue color indicate positive and negative correlation, respectively. (C) Each dot in the violin plots represents the correlation between a pair of genes. p values computed using Wilcoxon signed-rank test. (D) Number of proteins correlated with detrimental and protective module scores in each immune cell type in the Seattle cohort. (E) Heatmap of correlation between proteins in plasma samples and module 3 score in the Seattle cohort in immune cell types. See also Figure S6.

We selected 42 out of 96 genes with absolute effect size ^3^1 between the severe and mild trajectories (Table S5), resulting in 11, 13, 10, and 8 genes in modules 1, 2, 3, and 4, respectively. Module scores, defined as the geometric mean of expression of these reduced sets of genes in a given module, continued to be significantly positively (module 1, 2, and 3) and negatively (module 4) correlated with severity of viral infection (|r|^3^0.43, p < 2.23-16; Figure 6A), which suggested that genes within each module are correlated with each other. Indeed, we found most pairs of genes within each module were positively correlated, irrespective of their infection status (Figure 6B).

We found the correlation structure within each module changed depending on the presence and severity of infection. Pairwise correlations between genes in modules 1, 2, and 4 were significantly higher in patients with severe viral infection than HCs or patients with mild viral infection (p < 5e-05; Figure 6C). Pairwise correlations in module 2 were significantly lower in patients with mild infection compared to HCs (p = 3.6e-07; Figure 6C). In contrast, pairwise correlations between genes in module 3, which included genes involved in the protective host response, were significantly higher in patients with mild infection compared to HCs and those with severe infection (p = 5.7e-14; Figure 6C). Together, these results show that the genes within each module are expressed in a coordinated manner depending on the infection status and severity of infection.

### The protective host response modules are associated with IFN concentration in plasma proteome but decoupled from the IFN response in patients with severe viral infection

Recent reports have described higher expression of IFN-stimulated genes (ISGs) in patients with moderate SARS-CoV-2 infection than those with severe infection (Arunachalam et al., 2020). Therefore, we investigated whether this observation is generalizable to other viruses. Indeed, module 3 included three IFN-induced transmembrane (IFITM) genes (*IFITM1, IFITM2, IFITM3*), involved in the restriction of multiple viruses (Bailey et al., 2014), that were overexpressed in patients with viral infection and positively correlated with severity (Figure S6A). We also found several type I and II IFN receptors overexpressed during viral infection that positively correlated with severity, irrespective of the infecting virus (Figure S6A). In patients with mild viral infection, the distribution of correlations between IFITMs and genes in the protective response module 3 was significantly higher than in patients with severe viral infection or HCs (p ≤ 1e-06; Figure S6B). Further, the distribution of correlations between the type I and II IFN receptors and the protective response module 3 was not statistically different between HCs and patients with severe viral infection, but was significantly higher in patients with mild viral infection (p ≤ 0.03; Figure S6C).

We used 242 samples in the Seattle cohort for which both scRNA-seq and proteomic data were available to investigate whether detrimental or protective module scores are associated with IFN concentrations in plasma samples. Module 2 scores were not associated with any protein in any immune cell types, presumably because the majority of the genes in module 2 are specifically expressed in neutrophils, which are not included in PBMCs (Figure 6D). Scores for module 1, 3, and 4 were associated with several proteins in CD14+ monocytes. Module 3 score was associated with the several proteins in each immune cell type (Figures 6D and 6E) and had statistically significant positive correlation with IFN-gamma (IFNG) concentration across all cell types except CD16+ monocytes and proliferating T/NK cells, further suggesting that patients with higher module 3 scores may have better outcomes. Together, we found that expression of the ISGs increase with the severity of viral infection, but their correlation with the protective host response does not increase in patients with severe viral infection as much as those with mild viral infection. These results also show that higher module 3 score in several immune cell types is correlated with increased IFNG. Collectively, these results show a decoupling of the protective host response from the IFN response in patients with severe viral infection, irrespective of the virus.

### Host response-based module score improves classification of patients with severe and non-severe viral infection

**Figure 7:**
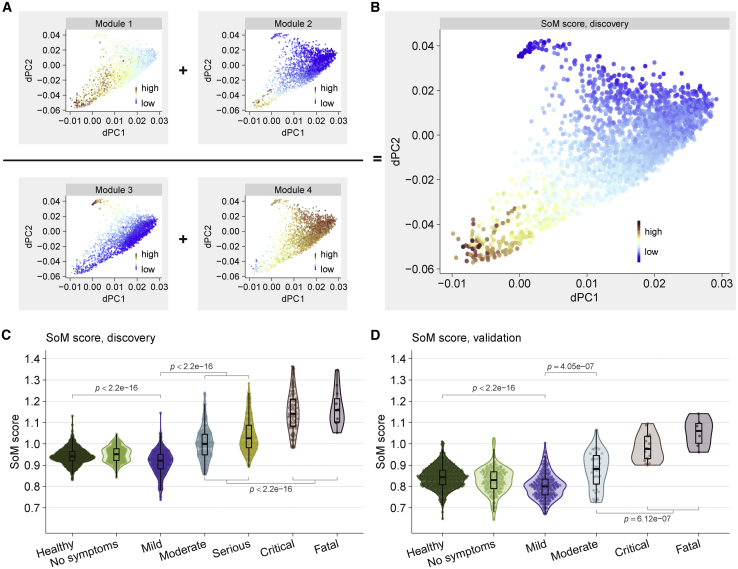
Host response modules improve classification of patients with severe and non-severe viral infection (A) Module scores and (B) the SoM score across the 3,183 dSpace samples. (C and D) The SoM score distinguishes mild and severe viral infection in the (C) discovery cohort and (D) validation cohort. Each point represents a sample. p values computed using Mann–Whitney U test. See also Figure S7.

Despite significant correlation with the severity, the MVS score cannot adequately distinguish severe and non-severe patients (Figures S7A and S7B). We hypothesized that incorporating the protective and detrimental host responses in a score would improve discrimination between severe and non-severe viral infection. Therefore, we defined the Severe-or-Mild (SoM) score of a sample as the sum of the scores for module 1 and 2 divided by the sum of the scores for module 3 and 4 (STAR Methods). The SoM score showed a more pronounced gradient between the severe and mild trajectories than any of the individual module scores (Figure 7B). The SoM score distinguished patients with mild infection from those with severe infection with AUROC^3^0.929 in the 3,183 samples from the discovery cohorts (Figure 7C, Figure S7C) and with AUROC > 0.98 in 5 independent validation cohorts comprised of 1,154 samples from patients infected with 4 different viruses (SARS-CoV-2, influenza, HRV, chikungunya) (Figure 7D, Figure S7D). In patients with non-severe viral infection, the SoM score distinguished those with mild infection from those with moderate infection with AUROC > 0.75 in discovery and validation datasets compared to the MVS score (AUROC < 0.63) (Figures S7A–S7D). In hospitalized patients with a viral infection, the SoM score also distinguished those with moderate infection from those with critical or fatal infection with higher accuracy than the MVS score (Figures S7A–S7D). Together, our results show that the protective and detrimental host response modules identified by trajectory analysis improve discrimination accuracy between patients with mild, moderate, and severe viral infection. Suppressing the detrimental host response modules or enhancing the protective host response modules could be therapeutic targets for host-directed broad-spectrum intervention in patients with severe viral infection.

## Discussion

The four viral pandemics since 2009 have underscored an urgent unmet need for creating generalizable diagnostic and therapeutic tools to enable faster deployment during infectious outbreaks. There is a strong impetus to develop virus-agnostic strategies that can be applied across multiple viruses. Here, we tested hypotheses that our previously described conserved host response to respiratory viral infections (Andres-Terre et al., 2015) represents biological mechanisms associated with severity that are distinct between patients with non-severe and severe outcomes, irrespective of infecting virus.

Although we had identified the MVS by analyzing respiratory viruses, it is generalizable across novel viruses, including SARS-CoV-2, chikungunya, and Ebola and across ages. Out of 37 cohorts, 12 cohorts consisted of 931 samples from children (<18 years), of which 643 were from children younger than 2 years. Our results demonstrate a conserved similarity in the dysregulation of the host immune response in patients with severe outcomes, which presents several opportunities the development of diagnostic and prognostic tests, identification of drug targets for host-directed broad-spectrum antiviral therapies, and drug repurposing to improve global pandemic preparedness for the pandemics that will invariably come in the future.

The SoM score using the 42-gene signature distinguished patients with a severe outcome from those with a non-severe outcome with very high accuracy. Arguably, the 42-gene signature is not optimal for clinical translation. However, given the high pairwise correlation between genes within each module, only a small subset of genes within each module could provide the same discriminatory power, allowing identification of a parsimonious gene signature. Trajectory analysis also suggests that the SoM score has the potential to predict the severity of outcome in patients with viral infection, though it needs to be tested in additional cohorts.

High pairwise correlations between genes in module 3 in patients with mild viral infection, irrespective of virus, suggest that a highly coordinated immune response between monocyte recruitment, interferon response, and cell death is associated with protection. Our results are consistent with recent observations that ISGs are strongly induced in patients with moderate SARS-CoV-2 infection compared to those with severe SARS-CoV-2 infection (Arunachalam et al., 2020; Hadjadj et al., 2020), and generalize to patients with non-severe versus severe infection, irrespective of the virus.

The genes in module 3 were more correlated with interferon-induced transmembrane proteins (IFITMs) in patients with mild infection compared to those with severe viral infection. IFITMs are involved in restricting viruses at various stages of the life cycle, including (1) blocking host cell entry by trapping virions in endosomal vesicles, (2) inhibiting viral gene expression and protein synthesis, and (3) disrupting viral assembly (Liao et al., 2019; Zhao et al., 2019). The lower correlations between the IFITMs and the genes in module 3 suggest that the IFN-induced response is “decoupled” from the protective response in during severe viral infection. Understanding the mechanisms underlying this decoupling could lead to targets for host-directed antiviral therapy.

Analysis of scRNA-seq in 3 independent cohorts and *in silico* deconvolution across 32 cohorts found increased HSPCs in patients with severe viral infection, irrespective of the virus, suggesting that emergency hematopoiesis is associated with increased risk of severity, possibly as the immune response fails to adequately respond to the virus. In contrast, we have previously shown reduced proportions of HSPCs in mild viral infection (Bongen et al., 2018), which may reflect the production of myeloid cells at the expense of the lymphoid compartment to replenish myeloid cells during infection (Takizawa et al., 2012). We found increased myeloid cells and reduced lymphoid cells in both scRNA-seq and deconvolution analysis, supporting a model where human HSPCs take an active role in the immune response by differentiating into myeloid cells, in line with our previous observation (Bongen et al., 2018).

In line with recent studies (Gatti et al., 2020; Hadjadj et al., 2020; Silvin et al., 2020; Zhou et al., 2020), we found decrease in CD16+ monocytes with increased severity. This suggests that reduced CD16+ monocytes in peripheral blood, possibly due to efflux to the site of infection in response to ongoing tissue damage or dysregulated cytokine sensing, is a conserved feature of the host response in severe viral infection across viruses and may have prognostic significance.

The mechanisms underlying the differential influence of CD14+ versus CD16+ monocytes and decoupling between the IFN-response and protective response remain unknown, though mounting evidence suggests dysfunctional myeloid cells in patients with severe viral infection. Dysfunctional CD14+ monocytes characterized by low expression of HLA-DR and high expression of alarmins are associated with severe COVID-19 infection (Schulte-Schrepping et al., 2020). However, our results show that it is a generalizable feature of severe outcome in other viral infections. Low HLA-DR expression in monocytes is a well-described marker of worse outcomes in sepsis and trauma (Hoffmann et al., 2017; Monneret et al., 2006; Venet et al., 2020). We found expression of *HLA-DPB1,* a MHC class II gene, in CD14+ monocytes was inversely correlated with severity of infection, whereas expression of alarmins (e.g., S100A9) in CD14+ monocytes was positively correlated with severity. Although overproduction of IL-6 mediates the low HLA-DR expression on CD14+ monocytes of patients with severe COVID-19 (Giamarellos-Bourboulis et al., 2020; Silvin et al., 2020), anti-IL6 therapy has been unsuccessful in showing improved outcomes in COVID-19 (Stone et al., 2020), highlighting the urgent need for elucidating the underlying mechanisms.

Our correlation analysis of protective module scores with plasma proteomic data in the patients with SARS-CoV-2 infection suggest dysfunctional myeloid cells in patients with severe outcome. Protective modules were significantly correlated with IFNG in antigen-presenting cells (CD14+ monocytes, cDCs, pDCs, B cells) and cytotoxic cells (NK cells, T cells). Combined with the observation that several class II HLA genes are downregulated in patients with severe outcome, it is possible that dysfunctional antigen-presenting cells are unable to orchestrate a subsequent adaptive immune response.

It is possible that factors such as genetics, comorbidities, and initial viral load, together or individually, cause an initial overactivation of monocytes, which leads to a sequence of CD14+ overactivation and secretion of inhibitors of HLA genes, further leading to a cascade of defective antigen presentation and T cell differentiation that fail to control ongoing infection. This may ultimately lead to the characteristic profile of severe disease: overactive dysfunctional myeloid and deficient lymphoid compartments amidst immunosuppression and a cytokine storm.

We found increased proportions of PMN- and M-MDSCs, and anti-inflammatory macrophages along with higher expression of their phenotypic and functional markers in patients with severe viral infection, but not in those with mild viral infection, irrespective of the virus. These results suggest that lower MDSCs in the early phase of infection is protective and provide strong evidence that, although increased PMN- and M-MDSCs may limit hyperinflammation during active viral infection, they may lead to a detrimental amplification of immunosuppression, irrespective of the virus. The modulation of monocyte responses, as reflected by gene expression, is compatible with the detrimental role of monocytes/macrophages in severe SARS-CoV-2 infection associated with respiratory dysfunction (Giamarellos-Bourboulis et al., 2020).

Among their immunosuppressive roles, MDSCs are known to suppress NK cell activity through arginase and ROS/RNS (Schrijver et al., 2019). Our trajectory and *in silico* deconvolution analyses, and scRNA-seq data found several NK cell-specific genes and the proportions of NK cells were negatively correlated with the severity of viral infection. We have previously shown that healthy individuals with lower expression of *KLRD1* are more likely to be infected when challenged (Bongen et al., 2018). A negative correlation between expression of *KLRD1* and the severity of viral infection, including SARS-CoV-2, further emphasizes that *KLRD1*-expressing NK cells may play a protective role following infection, irrespective of the infecting virus.

Taken together, our analyses offer a systems view of the immune state during viral infection and factors that mediate and predict progression to mild or severe outcomes, despite the heterogeneity and regardless of the infecting virus. We identified host response modules that could lead to new intervention strategies, including diagnostics for predicting patients at higher risk of severe outcomes, and broad-spectrum host-directed therapies for improved pandemic preparedness.

### Limitations of Study

Our analysis has two potential limitations. First, COCONUT co-normalization may have removed variability in immune responses of HCs of different ages and from different geographic regions. However, our results demonstrate that the effect of acute viral infection on the host immune response is substantially larger to overcome this heterogeneity. Second, it is possible that the MVS may be biased toward respiratory viral infections because it was discovered using respiratory viruses. However, the MVS score is also higher in patients with chikungunya or Ebola infection, suggesting that the MVS may be conserved across non-respiratory viruses too. It is also possible that the MVS only represents conserved host response to acute viral infection, and that chronic viruses such as hepatitis B or C, HIV, CMV, EBV, etc. may not evoke the same host response, which should be a focus of future studies.

## STAR★Methods

### Key resources table

**Table:** 

REAGENT or RESOURCE	SOURCE	IDENTIFIER
**Deposited data**		

RNA-seq dataset	Liu et al., 2017	accession# PRJNA352396
RNA-seq dataset	Soares-Schanoski et al., 2019	accession# PRJNA507472
RNA-seq dataset	Michlmayr et al., 2018	accession# PRJNA390289
RNA-seq dataset	Thair et al., 2020	accession# GSE152641
Microarray dataset	de Steenhuijsen Piters et al., 2016	accession# GSE77087
Microarray dataset	Liu et al., 2016	accession# GSE73072
Microarray dataset	Zhai et al., 2015	accession# GSE68310
Microarray dataset	Jaggi et al., 2018	accession# GSE68004
Microarray dataset	Heinonen et al., 2016	accession# GSE67059
Microarray dataset	Sweeney et al., 2015	accession# GSE66099
Microarray dataset	Wong et al., 2007, Wong et al., 2008, Wong et al., 2012	accession# GSE4607
Microarray dataset	Ramilo et al., 2007	accession# GSE6269
Microarray dataset	Hoang et al., 2014	accession# GSE61821
Microarray dataset	Davenport et al., 2014	accession# GSE61754
Microarray dataset	Parnell et al., 2012	accession# GSE40012
Microarray dataset	Mejias et al., 2013	accession# GSE38900
Microarray dataset	Wang et al., 2007	accession# GSE2729
Microarray dataset	Berdal et al., 2011	accession# GSE27131
Microarray dataset	Smith et al., 2014	accession# GSE25504
Microarray dataset	Bermejo-Martin et al., 2010	accession# GSE21802
Microarray dataset	Parnell et al., 2011	accession# GSE20346
Microarray dataset	Zaas et al., 2009	accession# GSE17156
Microarray dataset	Yu et al., 2019	accession# GSE117827
Microarray dataset	Dunning et al., 2018	accession# GSE111368
Microarray dataset	Rodriguez-Fernandez et al., 2017	accession# GSE103842
Microarray dataset	Tang et al., 2019	accession# GSE101702
Microarray dataset	Jong et al., 2016	accession# E-MTAB-5195
scRNA-seq dataset	Su et al., 2020	accession# E-MTAB-9357
scRNA-seq dataset	Arunachalam et al., 2020	accession# GSE155673
scRNA-seq dataset	Wilk et al., 2020	accession# GSE150728

**Software and Algorithms**		

R	R Core Team (2020)	www.r-project.org
gcRMA	Wu and Irizarry, 2020	https://www.bioconductor.org/packages/release/bioc/html/gcrma.html
MetaIntegrator	Haynes et al., 2017	https://cran.r-project.org/web/packages/MetaIntegrator/index.html
COCONUT	Sweeney et al., 2016b	https://cran.r-project.org/web/packages/COCONUT/index.html
Qualimap	García-Alcalde et al., 2012	http://qualimap.conesalab.org/
Salmon	Patro et al., 2017	https://combine-lab.github.io/salmon/
Tximport	Soneson and Robinson, 2018	https://bioconductor.org/packages/release/bioc/html/tximport.html
DESeq2	Love et al., 2014	https://bioconductor.org/packages/release/bioc/html/DESeq2.html
Alevin	Srivastava et al., 2019	https://combine-lab.github.io/salmon/
UMAP	McInnes et al., 2018	https://cran.r-project.org/web/packages/umap/index.html
Seurat	Satija et al., 2015	https://satijalab.org/seurat/
Trim Galore	Martiin, 2011	https://www.bioinformatics.babraham.ac.uk/projects/trim_galore/
SingleR	Aran et al., 2019	https://bioconductor.org/packages/release/bioc/html/SingleR.html
STAR	Dobin et al., 2013	https://github.com/alexdobin/STAR
immunoStates	Vallania et al., 2018	https://cran.r-project.org/web/packages/MetaIntegrator/index.html
tSpace	Dermadi et al., 2020	https://github.com/hylasD/tSpace

### Resource availability

#### Lead Contact

Further information and requests for resources, software, and data should be directed to and will be fulfilled by the Lead Contact, Purvesh Khatri (pkhatri@stanford.edu).

#### Materials Availability

This study did not generate new unique reagents.

#### Data and Code Availability

This study did not generate any unique datasets or code. All datasets, software, and algorithms used in this study are publicly available and listed in the Key Resource table.

### Quantification and Statistical Analysis

#### Dataset collection and preprocessing

We downloaded 26 gene expression datasets from the National Center for Biotechnology Information (NCBI) Gene Expression Omnibus (GEO), Sequence Read Archive (SRA), ArrayExpress, and European Nucleotide Archive (ENA), consisting of 4,780 samples from 34 independent cohorts derived from whole blood or peripheral blood mononuclear cells (PBMCs) (Table S1). We excluded all datasets used to discover the MVS previously to ensure all cohorts analyzed in the current study were independent. We defined a cohort as a comparable group of individuals within a dataset, where each dataset has a unique GEO identifier and may contain multiple cohorts. For example, the dataset GSE73072 contains seven cohorts of individuals challenged with one of three viruses. The samples in these datasets represented the biological and clinical heterogeneity observed in the real-world patient population, including HCs and patients infected with 16 different viruses with severity ranging from asymptomatic to fatal viral infection over a broad age range (0-90 years) (Figure 1A and Table S1). The samples were from patients enrolled across 18 different countries representing diverse genetic backgrounds of patients and viruses. We included technical heterogeneity in our analysis as these datasets were profiled using microarray and RNA sequencing (RNA-seq) from different manufacturers.

We renormalized all microarray datasets using standard methods when raw data were available from the GEO. We applied GC robust multiarray average (gcRMA) to arrays with mismatch probes for Affymetrix arrays. We used normal-exponential background correction and quantile normalization for Illumina, Agilent, GE, and other commercial arrays. We did not renormalize custom arrays and used preprocessed data as made publicly available by the study authors. We mapped microarray probes in each dataset to Entrez Gene identifiers (IDs) to facilitate integrated analysis. If a probe matched more than one gene, we expanded the expression data for that probe to add one record for each gene. When multiple probes mapped to the same gene within a dataset, we applied a fixed-effect model. Within a dataset, cohorts assayed with different microarray types were treated as independent.

#### Standardized severity assignment

For each dataset, we used the sample phenotypes as defined in the original publication. We manually assigned a severity category to each sample based on the cohort description for each dataset in the original publication as follows: (1) HCs – asymptomatic, uninfected healthy individuals, (2) asymptomatic or convalescents – afebrile asymptomatic individuals who tested positive for a virus or those fully recovered from a viral infection with completely resolved symptoms, (3) mild – symptomatic individuals with viral infection that were either managed as outpatient or discharged from the emergency department (ED), (4) moderate – symptomatic individuals with viral infection who were admitted to the general wards and did not require supplemental oxygen, (5) serious - symptomatic individuals with viral infection who were described as ‘severe’ by original authors, admitted to general wards with supplemental oxygen, or admitted to the intensive care unit (ICU) without requiring mechanical ventilation or inotropic support, (6) critical - symptomatic individuals with viral infection who were on mechanical ventilation in the ICU or were diagnosed with acute respiratory distress syndrome (ARDS), septic shock, or multiorgan dysfunction syndrome (MODS), and (7) fatal – patients with viral infection who died in the ICU.

For datasets that did not provide sample-level severity data (GSE101702, GSE38900, GSE103842, GSE66099, GSE77087), we assigned severity categories as follows. We categorized all samples in a dataset as “moderate” when either (1) > 70% of patients were admitted to the general wards as opposed to discharged from the ED, (2) < 20% of patients admitted to the general wards required supplemental oxygen, or (3) patients were admitted to the general wards and categorized as ‘mild’ or ‘moderate’ by the original authors. We categorized all samples in a dataset as “severe” when > 20% of patients had either (1) been admitted to the general wards and categorized as ‘severe’ by original authors, (2) required supplemental oxygen, or (3) required ICU admission without mechanical ventilation.

#### Viral challenge studies

GSE73072 included seven viral challenge studies that determined the infection status of a subject through reverse transcription PCR (RT-PCR) for a given virus (H1N1, H3N2, RSV, HRV) in longitudinally collected nasopharyngeal samples. In these studies, we assigned all baseline pre-challenge samples and subjects who never shed virus, as determined by RT-PCR, to the ‘healthy’ category. We assigned samples from infected subjects, defined as those who had virus detected in any of their nasopharyngeal samples, to one of three categories: (1) before infection - blood samples collected after challenge but before a virus was detected in a nasopharyngeal sample, (2) after infection - blood samples collected after the last nasopharyngeal sample in which a virus was detected, and (3) during infection - blood samples collected between the first and last nasopharyngeal sample in which a virus was detected.

#### COCONUT co-normalization

We used Combat CONormalization Using conTrols (COCONUT) for between-dataset normalization (Sweeney et al., 2016b). COCONUT allows for co-normalization of gene expression data without bias toward sample diagnosis by applying a modified version of the ComBat empirical Bayes normalization method (Johnson et al., 2007), which assumes a similar distribution between control samples. Briefly, healthy samples from each cohort undergo ComBat co-normalization without covariates, and the ComBat estimated parameters are computed for the healthy samples in each dataset. By applying these parameters to the non-healthy samples, all datasets keep the same background distribution while retaining the same relative distance between healthy and disease samples, which preserves the biological variability between the two groups within a dataset. We have previously shown that when COCONUT co-normalization is applied, housekeeping genes remain invariant across both conditions and cohorts, and each gene retains the same distribution across conditions within each dataset (Sweeney et al., 2016b).

#### MVS genes and score

We did not derive a *de novo* gene signature to represent the conserved host response to viral infection. Instead, we used our previously described 396-gene signature from peripheral blood (Andres-Terre et al., 2015). As previously described, we defined the MVS score of a sample as the difference between the geometric mean of the overexpressed genes and the geometric mean of the under-expressed genes in the MVS (Andres-Terre et al., 2015). Out of 396 genes in the MVS, 251 genes (111 over- and 140 under-expressed) were measured across all datasets. We used the 251 gene subset of the MVS in our analyses as the MVS score using either 251-gene or 396-gene signatures were highly correlated (data not shown).

We measured the correlation of the MVS score with viral infection severity using Spearman’s rank correlation coefficient. We used the Mann–Whitney U test (Wilcoxon rank-sum test) to compare MVS scores between two groups. We tested the trend of the MVS score along viral infection severity categories using the Jonckheere-Terpstra trend test.

#### RNA sequencing analysis

We obtained the raw reads for the Ebola (PRJNA352396) and chikungunya (PRJNA507472 and PRJNA390289) cohorts from the European Nucleotide Archive (ENA). We obtained the raw reads of the SARS-CoV-2 cohort from Inflammatix. We assessed trimmed Illumina adaptors and removed reads that were too short after adaptor trimming (less than 20 nt) with Trim Galore (v0.6.5). We then mapped the cleaned reads to the human genome (hg38) using STAR (v2.7.3) (Dobin et al., 2013). We performed additional quality control by checking the mapped reads with Qualimap (v.2.2.2) (García-Alcalde et al., 2012). To quantify gene expression, we obtained human transcriptome sequences from GENCODE site (v32), processed the cleaned reads with Salmon (1.2.1) (Patro et al., 2017) to get transcript-level expression, and summarized to gene-level expression using Tximport (v1.16.0) (Soneson and Robinson, 2018). Finally, we applied the variance stabilizing transformation from DESeq2 (v1.26.0) (Love et al., 2014) to normalize gene expression for downstream analysis and visualization.

#### Detection of viral reads in RNA-seq data

We obtained genome sequences of 501 human viruses from the NCBI virus database (accessed on April 19, 2020). We concatenated viral sequences with the list human transcriptome sequences and built a decoy-aware index using Salmon. We mapped the reads to the concatenated index using Salmon with the selective-alignment algorithm, which, together with the decoy-aware index, mitigates potential spurious mapping of reads arising from unannotated human genomic loci and reduces false positives. We extracted reads mapped to viral genomes and filtered them to remove secondary alignments and paired-end reads with only one mate mapped. We also checked the reads with NCBI Nucleotide BLAST to ensure viral origin. We normalized the viral read counts by the total number of sequencing reads of each sample. We measured the correlation between the MVS score and viral read counts using Pearson correlation coefficient.

#### Analysis of single-cell RNA-seq data

We downloaded scRNA-seq data of the Stanford cohort from NCBI GEO (Wilk et al., 2020). We also obtained the scRNA-seq data for the Atlanta cohort (Arunachalam et al., 2020) and the Seattle cohort (Su et al., 2020). We processed raw scRNA-seq data with Alevin (v1.2.1) (Srivastava et al., 2019) to get the read count matrices. We performed quality control, normalization, dimension reduction, UMAP projection, and Shared Nearest Neighbors clustering on the three datasets with Seurat (Satija et al., 2015). Then we applied the Seurat integration workflow to integrate the three datasets using Reciprocal PCA analysis. Cell type was annotated with SingleR inference (Aran et al., 2019), cell type markers, and annotations from the original publications.

#### In silico cellular deconvolution using immunoStates and multi-cohort analysis of estimated cellular proportions

We performed *in silico* cellular deconvolution using immunoStates as a basis matrix with support vector regression to estimate proportions of 25 immune cell subsets in each sample (Vallania et al., 2018). To investigate changes in the immune cell proportions between patients with different severity of viral infection, we conducted three multi-cohort analyses using MetaIntegrator R package (Haynes et al., 2017) between samples from the following categories: 1) subjects with non-severe viral infection (severity categories ‘mild’ and ‘moderate’) versus HCs, 2) subjects with severe viral infection (severity categories ‘serious’, ‘critical’, and ‘fatal’) versus HCs, and 3) subjects with severe viral infection versus subjects with non-severe viral infection. We combined effect sizes across studies using a random-effects inverse variance model. For each meta-analysis, we calculated the change in proportions for each immune cell type between groups in each cohort as the Hedges’ g effect size (ES). We corrected p values for multiple hypotheses testing using the Benjamini-Hochberg correction to obtain the false discovery rate (FDR). We used a threshold of FDR < 20% and representation in a minimum of 5 studies in conjunction with leave-one-out analysis to identify immune cell types with increased or decreased proportions between groups. Individual samples that met the following criteria were excluded: non-viral infection, non- HCs, and one sample from PRJNA252396 (SRR4888654) which had the same expression value for all 317 genes. Datasets with less than two samples in each of the compared groups were excluded from meta-analysis. Individual samples that met the following criteria were excluded: non-viral infection, non-healthy control, and one sample from PRJNA252396 (SRR4888654) which had the same expression value for all 317 genes. Datasets with less than two samples in each of the compared groups were excluded from meta-analysis.

#### Trajectory inference analysis

We co-normalized 1674 samples from 21 cohorts in 19 datasets with 1509 samples from four independent challenge studies using COCONUT. Each challenge study inoculated healthy volunteers with one of four viruses (HRV, RSV, H1N1, and H3N2). We adapted tSpace, a method for identifying cellular differentiation trajectories using scRNA-seq data (Dermadi et al., 2020), to identify disease trajectories using bulk transcriptome profiles. We refer to the adaption to bulk transcriptome data as disease space (dSpace), although the core method remains identical to tSpace. The tSpace algorithm has three steps: (1) calculation of a set of sub-graphs, (2) calculation of the trajectory space matrix across the sub-graphs, and (3) visualization. In the first step, we calculated a set of sub-graphs keeping L out of K nearest neighbors in a KNN graph. The user defines the number of sub-graphs (G), neighborhood size (K), and how many nearest neighbors are preserved in the sub-graphs (L). The second step computes a *trajectory space* distance matrix using a modified Dijkstra algorithm that implements waypoints (WP) to exponentially weigh and refine distances. The final *trajectory space* matrix is a dense matrix in which each sample is a row, and calculated trajectories are columns. The number of trajectories (T > 150) is user-defined and very robust across a wide dynamic range. Finally, we visualize the samples and their relationships in trajectory space using PCA or UMAP.

We used the following parameters for the dSpace analysis: G = 5, K = 65, L = 49, T = 500, WP = 20. We used Pearson correlation as the metric for computing distance between two samples. We fitted a principal line through data visualized in the first two components of tSpace (tPC1, tPC2) using the princurve R package. Princurve calculates lambda, an arc length distance for each data point, which we used to align subjects along the isolated trajectory. Furthermore, the covariance matrix of the transposed trajectory matrix (covariance mapping) coupled with the hierarchical clustering identified clusters of patients with shared trajectory space. The covariance matrix of the transposed trajectory matrix allows identification of patients that belong to diverging trajectories, and hierarchical clustering of covariance matrix allowed us to group patients that are in severe and non-severe branches, thus enabling isolation of both branches. Each of the determined clusters is a reflection of the position of patients in the trajectory space. Hierarchical clustering was calculated using hclust and Dist R functions with “euclidean” and “complete” parameters.

Severe and non-severe trajectories shared 1020 samples from the healthy and no symptoms categories. Therefore, we aligned them using dynamic time warping (dtw R package) and split them into 4 stages. All 251 genes and the fitted trajectory (lambda value) were used for alignment. We applied a permutation test (Efron and Tibshirani, 2002) for each of the 4 stages and identified 96 genes that were differentially expressed within the same stage between the two severity branches.

#### Statistical analysis of trajectories identified with dSpace

We applied a permutation test (Efron and Tibshirani, 2002) for each of the 4 stages and identified 96 genes that were differentially expressed within the same stage between the two severity branches. In our testing we used 1000 permutations, and for significance FDR < 0.001 and |effects size| > 0.3. We performed data analysis using R.

#### Calculation of the SoM score

The Severe or Mild (SoM) score is a 42-gene model that utilizes the expression of genes from the 4 gene modules to distinguish between severe and mild viral infection. For each sample, we compute the geometric mean of the expression of genes from each module. Then, we calculate a score by taking the sum of the geometric means of modules 1 and 2 and dividing that by the sum of the geometric means of modules 3 and 4, as shown in the following equation:

(Equation1) $$SoM\;score=\frac{{\left(\prod_{gene\;\varepsilon \;Module1}\;x_{i}\left(gene\right)\right)}^{\frac{1}{\left|\right|Module1\left|\right|}}+{\left(\prod_{gene\;\varepsilon \;Module2}\;x_{i}\left(gene\right)\right)}^{\frac{1}{\left|\right|Module2\left|\right|}}}{{\left(\prod_{gene\;\varepsilon \;Module3}\;x_{i}\left(gene\right)\right)}^{\frac{1}{\left|\right|Module3\left|\right|}}+{\left(\prod_{gene\;\varepsilon \;Module4}\;x_{i}\left(gene\right)\right)}^{\frac{1}{\left|\right|Module4\left|\right|}}}$$

#### Correlation of cell proportions, MVS score, and SoM score with severity

We measured the correlation of the cell proportions or the score with viral infection severity using Spearman’s rank correlation coefficient. We used the Mann–Whitney U test (Wilcoxon rank-sum test) to compare the cell proportions or the score between two groups. We tested the trend of the cell proportions or the score along viral infection severity categories using the Jonckheere-Terpstra trend test. We measured the correlation between the 251 and 396 gene versions of the MVS using Pearson’s correlation coefficient. Data analysis was performed using R.

#### Module score association with plasma proteomics

The association between modules scores and plasma proteomics data from the Seattle cohort were calculated using multivariate generalized estimating equations to account for repeated samples per patient and adjust for covariates (age, sex, BMI, race/ethnicity). The p values were adjusted using Bonferroni correction. Data analysis was performed using R.

## Supplemental information

Document S1. Figures S1–S7 and Data S1

Table S1. Overview of datasets and analysis, related to Figure 1

Table S2: Samples in three cohorts of scRNA-seq, related to Figure 2

Table S3: Number of cohorts and samples used in each multi-cohort analysis of estimated cell proportions, related to Figure 2 and Figure 5

Table S4. Summary effect sizes, p values, and FDRs of significantly different immune cell subtypes from multi-cohort analysis of relative immune cell proportions comparing various viral infection severity levels, related to Figure 2 and Figure 5

Table S5: List of 96 genes identified by dSpace analysis and their correlation with severity, effect size between severe and non-severe patients, and FDR, related to Figure 5

Document S2. Article plus supplemental information
