# Supplementary material for: Multi-cohort analysis of host immune response identifies conserved protective and detrimental modules associated with severity across viruses
Source: Immunity. 2021 Apr 13;54(4):753–768.e5. doi: 10.1016/j.immuni.2021.03.002 (PMC7988739; doi:10.1016/j.immuni.2021.03.002)
Supplement: Table S3: Number of cohorts and samples used in each multi-cohort analysis of estimated cell proportions, related to Figure 2 and Figure 5 [file mmc4.pdf]

TableS3: Number of cohorts and sample counts used in each meta-analysis of estimated relative cell proportions.

Meta-analysis	Number of dataset subsets	Number of independent cohorts	Total number of samples	Number of samples per group
Non-severe viral infection vs healthy controls	25	23	3666	1092 non-severe<br>2574 healthy
Severe viral infection vs healthy controls	15	14	816	399 severe<br>417 healthy
Severe viral infection vs non-severe viral infection	9	8	610	254 severe<br>356 non-severe
Total number of dataset subsets:	37			
Total number of independent cohorts:	32			
Total number of unique samples:	4357			
