# Supplementary material for: Multi-cohort analysis of host immune response identifies conserved protective and detrimental modules associated with severity across viruses
Source: Immunity. 2021 Apr 13;54(4):753–768.e5. doi: 10.1016/j.immuni.2021.03.002 (PMC7988739; doi:10.1016/j.immuni.2021.03.002)
Supplement: Table S4. Summary effect sizes, p values, and FDRs of significantly different immune cell subtypes from multi-cohort analysis of relative immune cell proportions comparing various viral infection severity levels, related to Figure 2 and Figure 5 [file mmc5.pdf]

TableS4: Summary effect sizes, p-values, and FDRs of significantly different immune cell subtypes from meta-analyses of relative immune cell proportions comparing various viral infection severity levels.

	Immune cell type	Summary effect size	Effect size p-value	Effect size FDR	Number of studies
**Non-severe viral infection vs healthy controls**	CD14+ monocytes	1.12	1.04E-22	1.30E-21	24
	Total monocytes	1.10	1.21E-13	4.33E-13	25
	M1 macrophages	0.88	1.23E-15	6.16E-15	24
	Plasma cells	0.39	1.65E-03	3.74E-03	24
	Plasmacytoid DCs	0.31	1.30E-03	3.24E-03	24
	Neutrophils	0.18	4.20E-02	7.01E-02	25
	Total granulocytes	0.10	2.93E-01	4.07E-01	25
	CD16+ monocytes	0.05	6.37E-01	7.24E-01	25
	Mast cells	0.05	4.79E-01	6.30E-01	17
	γδ T cells	0.04	6.03E-01	7.17E-01	15
	M0 macrophages	0.04	6.77E-01	7.36E-01	16
	Memory B cells	0.01	8.60E-01	8.60E-01	16
	Hematopoietic progenitors	-0.02	7.38E-01	7.69E-01	22
	Conventional DCs	-0.10	5.63E-01	7.04E-01	9
	Basophils	-0.10	1.18E-01	1.85E-01	25
	Total NK cells	-0.11	2.68E-01	3.94E-01	25
	Eosinophils	-0.28	6.42E-03	1.34E-02	21
	M2 macrophages	-0.48	1.08E-03	3.00E-03	25
	CD8+ αβ T cells	-0.58	3.13E-14	1.31E-13	25
	CD4+ αβ T cells	-0.61	3.82E-09	1.20E-08	25
	Total B cells	-0.75	5.02E-17	4.19E-16	25
	Naïve B cells	-0.80	1.24E-25	3.09E-24	25
	Total T cells	-0.82	2.90E-16	1.81E-15	25
**Severe viral infection vs healthy controls**	M1 macrophages	1.36	9.26E-12	5.12E-11	15
	Neutrophils	1.24	1.65E-17	4.12E-16	15
	Total granulocytes	1.16	7.90E-15	9.88E-14	15
	Plasma cells	0.93	1.66E-05	4.16E-05	15
	CD14+ monocytes	0.90	1.57E-10	6.56E-10	15
	Hematopoietic progenitors	0.85	4.69E-04	7.33E-04	15
	M2 macrophages	0.63	2.25E-06	7.02E-06	15
	Total monocytes	0.15	5.02E-01	5.23E-01	15
	Memory B cells	0.13	5.89E-01	5.89E-01	8
	Conventional DCs	-0.48	2.01E-03	2.95E-03	4
	Basophils	-0.30	2.56E-02	2.91E-02	15
	Eosinophils	-0.43	7.50E-03	1.04E-02	11
	Total B cells	-0.47	2.00E-02	2.50E-02	15
	M0 macrophages	-0.51	1.04E-02	1.37E-02	9

	CD8+ αβ T cells	-0.73	1.02E-05	2.84E-05	15
	Total NK cells	-0.85	3.95E-05	8.97E-05	15
	CD4+ αβ T cells	-0.86	1.08E-04	1.80E-04	15
	γδ T cells	-0.89	4.32E-05	9.01E-05	5
	Naïve B cells	-1.12	4.07E-14	3.39E-13	15
	CD16+ monocytes	-1.16	1.44E-08	5.13E-08	15
	Total T cells	-1.37	1.02E-11	5.12E-11	15
	Mast cells	-1.49	2.10E-02	2.50E-02	5
**Severe viral infection vs non-severe viral infection**	Total granulocytes	1.01	1.82E-08	1.51E-07	9
	Neutrophils	0.99	6.94E-08	4.33E-07	9
	M2 macrophages	0.76	2.50E-10	3.12E-09	8
	Hematopoietic progenitors	0.43	1.17E-02	3.38E-02	9
	M1 macrophages	0.29	8.62E-02	1.54E-01	9
	Plasma cells	0.23	3.29E-01	5.15E-01	9
	CD14+ monocytes	0.10	2.69E-01	4.48E-01	9
	Memory B cells	0.08	4.60E-01	6.39E-01	6
	Plasmacytoid DCs	0.06	6.30E-01	7.50E-01	9
	M0 macrophages	-0.02	8.66E-01	8.66E-01	7
	Basophils	-0.02	8.51E-01	8.66E-01	9
	Mast cells	-0.04	8.08E-01	8.66E-01	3
	Total B cells	-0.08	5.42E-01	6.77E-01	9
	Eosinophils	-0.14	5.06E-01	6.66E-01	7
	CD8+ αβ T cells	-0.16	3.70E-01	5.44E-01	9
	Naïve B cells	-0.27	1.22E-02	3.38E-02	9
	Conventional DCs	-0.30	1.72E-02	3.91E-02	5
	γδ T cells	-0.42	1.52E-02	3.81E-02	3
	Total T cells	-0.45	8.25E-02	1.54E-01	9
	Total monocytes	-0.50	1.61E-05	5.76E-05	9
	CD4+ αβ T cells	-0.52	4.61E-02	9.60E-02	9
	CD16+ monocytes	-0.88	6.92E-19	1.73E-17	9
	Total NK cells	-1.03	2.70E-07	1.13E-06	9
