# Supplementary material for: Multi-cohort analysis of host immune response identifies conserved protective and detrimental modules associated with severity across viruses
Source: Immunity. 2021 Apr 13;54(4):753–768.e5. doi: 10.1016/j.immuni.2021.03.002 (PMC7988739; doi:10.1016/j.immuni.2021.03.002)
Supplement: Table S5: List of 96 genes identified by dSpace analysis and their correlation with severity, effect size between severe and non-severe patients, and FDR, related to Figure 5 [file mmc6.pdf]

TableS5: 96 genes identified from trajectory analysis

gene	Module	Correlation with severity	Severe vs non-severe effect size	Severe vs non-severe FDR
TXN	1	0.49	1.65	2.00E-74
ORM1	1	0.16	1.58	4.77E-72
PFKFB4	1	0.43	1.50	4.69E-62
SLPI	1	0.36	1.44	2.71E-65
BCL6	1	0.35	1.32	4.41E-56
NQO2	1	0.23	1.26	8.14E-49
AQP9	1	0.41	1.19	4.70E-50
ANXA3	1	0.52	1.07	7.38E-42
DOK3	1	0.44	1.07	5.89E-42
KLHL2	1	0.20	1.01	1.09E-38
TYK2	1	-0.02	1.01	1.31E-35
TLN1	1	0.16	0.88	4.78E-31
ACSL1	1	0.38	0.80	2.31E-29
SRGN	1	0.38	0.69	1.04E-20
GRN	1	0.51	0.58	3.52E-16
ADM	1	0.56	0.53	3.20E-16
NUCB1	1	0.38	0.48	1.16E-11
TLR2	1	0.25	0.38	6.12E-07
BCL2A1	1	0.33	0.18	6.69E-04
CAMP	2	0.40	1.54	1.32E-75
LCN2	2	0.35	1.40	7.32E-65
DEFA4	2	0.29	1.35	1.44E-57
CTSG	2	0.28	1.31	1.30E-66
CEACAM8	2	0.31	1.30	1.31E-57
BCAT1	2	0.41	1.26	1.08E-55
BTBD7	2	0.22	1.23	1.06E-45
KIF15	2	0.43	1.19	2.41E-54
AZU1	2	0.27	1.17	1.74E-58
CEP55	2	0.45	1.16	2.76E-58
PRC1	2	0.37	1.16	2.30E-56
HMMR	2	0.46	1.06	1.15E-51
BCL2L11	2	0.39	1.02	2.58E-39
CDT1	2	0.42	0.96	1.50E-40
TCEAL9	2	0.35	0.90	1.62E-41
OLR1	2	0.33	0.89	1.47E-42
TRIP13	2	0.39	0.87	1.07E-34
ELL2	2	0.41	0.74	7.34E-25
SOCS6	2	0.23	0.69	8.49E-21

IGFBP2	2	0.26	0.66	2.89E-22
ATP8B4	2	0.48	0.61	8.36E-18
KIF23	2	0.47	0.31	1.09E-07
UBE2L6	3	0.29	-1.84	9.42E-79
CASP7	3	0.09	-1.44	1.46E-58
OASL	3	0.54	-1.32	1.52E-54
TMEM123	3	0.16	-1.32	1.08E-51
VRK2	3	0.25	-1.26	1.46E-49
NAPA	3	0.16	-1.09	1.64E-41
CCL2	3	0.26	-1.09	1.30E-66
MAFB	3	0.32	-1.09	1.88E-38
VAMP5	3	0.46	-1.07	1.15E-38
ATG3	3	0.18	-1.01	4.36E-38
FAM8A1	3	0.08	-0.89	1.22E-27
LAPTM4A	3	-0.12	-0.84	3.61E-29
ANXA2	3	0.13	-0.80	2.18E-26
SSR2	3	-0.34	-0.80	3.69E-21
IFITM3	3	0.59	-0.41	6.10E-07
POMP	3	0.31	-0.36	6.49E-11
IFITM1	3	0.54	-0.29	1.15E-03
CREG1	3	0.33	-0.24	9.71E-05
SCAND1	3	0.20	-0.04	4.70E-01
RAD23B	3	-0.04	0.18	5.42E-01
H1-0	3	0.45	0.51	2.97E-12
IFITM2	3	0.49	0.77	2.58E-26
FURIN	3	0.17	0.88	5.33E-30
HLA-DPB1	4	-0.41	-1.82	1.64E-81
SMYD2	4	-0.39	-1.39	1.67E-57
SIDT1	4	-0.44	-1.39	2.30E-61
TRIB2	4	-0.29	-1.35	1.20E-57
DOK2	4	-0.26	-1.24	2.23E-52
KLRB1	4	-0.49	-1.13	2.03E-49
EXOC2	4	-0.22	-1.11	2.40E-45
BUB3	4	-0.40	-1.11	8.75E-42
KLRG1	4	-0.38	-0.97	1.14E-32
PRSS23	4	-0.33	-0.96	1.90E-30
KLRD1	4	-0.27	-0.89	7.73E-28
PRF1	4	-0.24	-0.89	8.60E-30
USP11	4	-0.40	-0.85	7.63E-30
BANF1	4	-0.21	-0.84	1.65E-25
CHMP7	4	-0.47	-0.84	3.29E-28
RBM15B	4	-0.37	-0.81	3.64E-22

MAP3K4	4	-0.42	-0.81	1.45E-25
ITGB7	4	-0.25	-0.75	2.12E-22
EPHX2	4	-0.46	-0.69	1.46E-18
IL7R	4	-0.40	-0.69	2.09E-19
DDB1	4	-0.41	-0.67	3.59E-18
LRBA	4	-0.41	-0.64	1.63E-16
TRAF5	4	-0.54	-0.60	2.18E-13
ARHGAP45	4	-0.38	-0.59	2.09E-14
CCR7	4	-0.40	-0.57	5.13E-12
PITPNC1	4	-0.51	-0.54	5.99E-12
TRAF3IP3	4	-0.38	-0.54	7.38E-13
LTBP3	4	-0.43	-0.51	6.66E-11
FBLN5	4	-0.30	-0.51	4.31E-11
TRIM28	4	-0.36	-0.51	2.64E-12
PIK3R1	4	-0.33	-0.32	1.87E-05
EZH1	4	-0.29	0.14	2.29E-01
